# Autism spectrum: parents’ perspectives reflecting the different needs of different families

**DOI:** 10.1186/s12887-024-04912-x

**Published:** 2024-07-09

**Authors:** Nadja K. Battanta, Oskar G. Jenni, Christina Schaefer, Michael von Rhein

**Affiliations:** 1 Child Development Center, University Children’s Hospital Zurich, Zurich, Switzerland; 2 Children’s Research Center, University Children’s Hospital Zurich, Zurich, Switzerland; 3 University of Zurich, Zurich, Switzerland

**Keywords:** Autism spectrum disorder, Parents needs, Challenges, Care gaps, Parental stress, Financial counselling

## Abstract

**Background:**

Parents of children on the autism spectrum often face great challenges in the care of their child. Early support tailored to families’ individual needs is therefore crucial for the development and quality of life of both children on the autism spectrum and their families. However, to date it is unclear whether the support available meets the parents’ needs.

**Study Aim:**

To investigate how the system of care, support, and therapies for children on the autism spectrum is perceived by their parents.

**Method:**

A total of 57 parents of Swiss children on the autism spectrum participated in an online survey, and 20 of them participated in additional semi-structured interviews.

**Results:**

We found that parents of children on the autism spectrum may face substantial challenges and that social support is essential. Two thirds of the participating parents reported a long and difficult diagnostic process as challenging, and 60% expressed their need for closer follow-up after diagnosis and more support. Only one third of the parents stated that they manage their everyday lives well, whereas 17.5% felt exhausted, and more than half of the parents responded that they felt challenged. One fifth indicated that they had poor family support, and half reported substantial financial challenges. At the same time, most families also emphasize how important their neurodivergent children are to the family`s life together.

**Conclusion:**

It is important that primary pediatricians not only initiate the diagnostic process, but also assess the different needs of the different family independent of the diagnosis and, if necessary, initiate adequate measures or guide parents to institutions in charge. Parents who do not actively express their individual needs should nevertheless be advised about support services, including financial counseling. The positive aspects mentioned by families can be emphasized and used as resources to improve their quality of life.

**Supplementary Information:**

The online version contains supplementary material available at 10.1186/s12887-024-04912-x.

## Introduction

Autism spectrum development (ASD) is a heterogeneous neurodevelopmental condition [1] characterized by challenges in reciprocal social interaction and social communication and neurodivergence in behavior patterns, interests, and activities. These traits often become evident in early childhood [2, 3].

Despite improvements in diagnostic practice, there is still a shortage of diagnostic services, leading to an overload of diagnostic centers and a lengthy diagnostic process in many regions [4]. This delay prolongs the sometimes stressful situation of uncertainty for parents and limits their access to appropriate support [5].

In recent years, interest has grown in developing effective interventions for young children on the autism spectrum. At preschool age, various interventions are available for neurodivergent children, including special education and speech therapy [6]. In Switzerland, intensive intervention programs are available for children on the autism spectrum and their families, including behavioral, developmental, and relationship-based programs. However, while the number of children on the autism spectrum and the desire for specific support has increased in recent years, specialized intervention programs for children with an early diagnosis are not sufficiently or comprehensively available [5, 7, 8].

The care of a child on the autism spectrum can be demanding and can have far-reaching effects on family life that can also lead to severe stress for parents [9–11]. Studies have shown that parents of children on the autism spectrum sometimes experience significantly higher levels of stress than do parents of children with developmental delays, other forms of neurodivergence such as Down syndrome, and neurotypical children [12–15]. The challenges experienced by these families can lead to limitations in physical and psychological well-being, resulting in elevated stress levels and high rates of depression and anxiety disorders in parents [12, 13, 16]. Moreover, financial burdens are often cited as a significant stressor for families with children on the autism spectrum. This is due in part to the high cost of many interventions, special schools, and other services, which are often at least partially funded by parents themselves [11, 17]. The families often also experience financial losses due to the high care requirements of an autistic child, which often result in one parent having to reduce their workload or even give up their job [11, 17–19].

The perspectives of families with children on the autism spectrum on their care and everyday life have so far only been addressed by research to a limited extent. During the past decade, several studies have shed light on various aspects of parents’ perceptions and needs throughout the diagnostic process as well as in service access and delivery [20, 21]. However, results on parenting stress and family experiences vary widely. Most studies are based on a limited number of interviews, and almost all are from Australia or North America [22, 23]. The aim of our research project was therefore to assess the type and extent of care provided to families with children on the autism spectrum and their specific support needs in a sample of affected families in a resource-rich European country with a very well-developed social system. Our study aimed to describe the care situation, including diagnostic workup, therapeutic measures, and leisure activities, from the families’ perspective by asking them about their daily experiences. Our research question was: In which areas do parents of a child on the autism spectrum perceive the most important gaps in care and support in the metropolitan area of Zurich, Switzerland?

## Methods

The research question was addressed with a mixed-method research design that combined quantitative and qualitative data. The experiences and needs of families were explored through a survey and semi-structured interviews. The survey results were complemented by the insights gained from the individual interviews.

### Participants and recruitment procedures

The Canton of Zurich, Switzerland, which has a population of 1.5 million, has two units for special needs education (USNEs), which determine the individual support needs of preschool children. All children referred for early interventions are evaluated by these units. The USNEs are responsible for assessing individual early intervention needs, approving registration for special educational or speech therapy intervention, and if indicated, allocating children to suitable measures. After approval by the USNEs, the costs for the interventions are covered by the Cantonal authorities without the families incurring any additional costs. We began our study recruitment by screening the USNE database for the keywords ASD, autism, Asperger syndrome, and autism diagnostic observation schedule (ADOS). Inclusion criteria required that children between birth and 5 years of age were diagnosed with autism spectrum development between 2014 and 2017 and had been enrolled for early special needs education. Additionally, knowledge of German sufficient to complete the survey was required at least in one parent. A total of 147 families who met these criteria were found in the database and invited to participate in the study. Of those invited, 57 families completed the questionnaire; 44 preferred the paper version and 13 used the online version (n: 57, participation rate: 38.8%). From this total, 20 (13.6%) candidates were selected and recruited for interview.

### Assessment tools

The first part of the study used a questionnaire developed by the authors with 70 quantitative questions about the child’s development, therapies, and the family’s current living situation. It included questions about the developmental domain in which concerns were noticed by the parents, such as speech, motor skills, cognition, and social and emotional competences, and when they first noticed them. We also asked for the child’s age at first contact with a specialist due to these challenges, their age at diagnosis, and about the parents’ subjective perceptions of family support and coping with everyday life (rated on a 3-point scale). In addition, questions were asked about the additional costs arising from the need for support and the influence of this on the parents’ professional activities. The semi-structured interviews with parents consisted of qualitative questions about the development of the child, experience with the diagnostic workup, therapeutic measures, and the daily life of the family. In all, 17 interviews were conducted over a 3-month period via videoconferencing systems and 3 by telephone. After parental consent was obtained, the interviews were recorded and subsequently transcribed and anonymized. The questionnaire and the interview template are provided in the supplement. The quotes taken from the interviews and used for the analysis were translated from German into English by the authors with the aim of reflecting the statements of the interviewees as closely as possible. The questionnaire and the interview template are provided in the supplement.

### Data analysis

After entering all data into a research database (REDCap©), we analyzed the quantitative data using IBM-SPSS© version 27. We calculated descriptive statistics, frequencies, percentages, and distributions, which we present here in graphs and tables. For the qualitative analysis, we transcribed the interviews by verbatim transcription and anonymized all identifying information to ensure participant confidentiality [24]. The interviews were transcribed, coded, and analyzed using MAXQDA 2020©, a qualitative data analysis program. To obtain systematic qualitative evaluation, Mayring’s qualitative content analysis method was employed [24, 25]. A system of theory-based categories was developed to identify aspects that need to be extracted from the interview material [24]. The categories were formulated deductively from theoretical prior knowledge and supplemented with inductive categories derived from the material. This was done using a summarizing content analysis [24, 25]. Once the categories had been defined, anchor examples were created from passages in the text that matched the content. The material was coded using this category system by categorizing all text passages that fitted the content of a category [25]. Further analysis was conducted by inductive or deductive formation of subcategories and by quantitative analyses of, for example, frequency of categories. The categories used for the coding are provided in the supplement.

## Results

Demographics of the participating families (*n* = 57) are summarized in Table 1.

**Table 1:** Demographics

Demographics	Survey [*n* = 57]		Interview [*n* = 20]	
	*n*	%	*n*	%
**Gender of child with ASD**				
Male	50	87.7	17	85.0
Female	7	12.3	3	15.0
**Age of child with ASD [years]**				
Mean +/- SD	8.9 +/- 1.3		9.0 +/- 1.3	
Minimum	5.7		6.6	
Maximum	12.0		11.7	
**Age of mother [years]**				
Mean +/- SD	39.8 +/- 5.1		40.2 +/- 4.7	
Minimum	29		31	
Maximum	52		50	
**Age of father [years]**				
Mean +/- SD	43.1 +/- 5.9		43.0 +/- 5.7	
Minimum	31		32	
Maximum	56		53	
**Origin mother**				
Switzerland	12	21.1	8	40.0
Europe	20	35.1	8	40.0
Africa	6	10.5	0	0.0
Asia	14	24.6	2	10.0
North and South America	5	8.8	2	10.0
**Origin father**				
Switzerland	17	29.8	11	55.0
Europe	21	36.8	8	40.0
Africa	4	7.0	0	0.0
Asia	12	21.1	1	5.0
North and South America	2	3.5	0	0.0
Missing	1	1.8	0	0.0
**Highest school degree mother**				
Compulsory school	7	12.3	1	5.0
Apprenticeship	15	26.3	5	25.0
High school	7	12.3	2	10.0
Higher technical school	5	8.8	4	20.0
University of Applied Sciences	1	1.8	0	0.0
University degree	21	36.8	8	40.0
No school-leaving qualification	1	1.8	0	0.0
**Highest school degree father**				
Compulsory school	4	7.0	0	0,0
Apprenticeship	11	19.3	5	25.0
High school	2	3.5	0	0,0
Higher technical school	8	14.0	4	20.0
University of Applied Sciences	7	12.3	4	20.0
University degree	19	33.3	7	35.0
No school-leaving qualification	3	5.3	0	0.0
Missing	3	5.3	0	0.0

### Development of the child and diagnostic workup

**Fig. 1:**
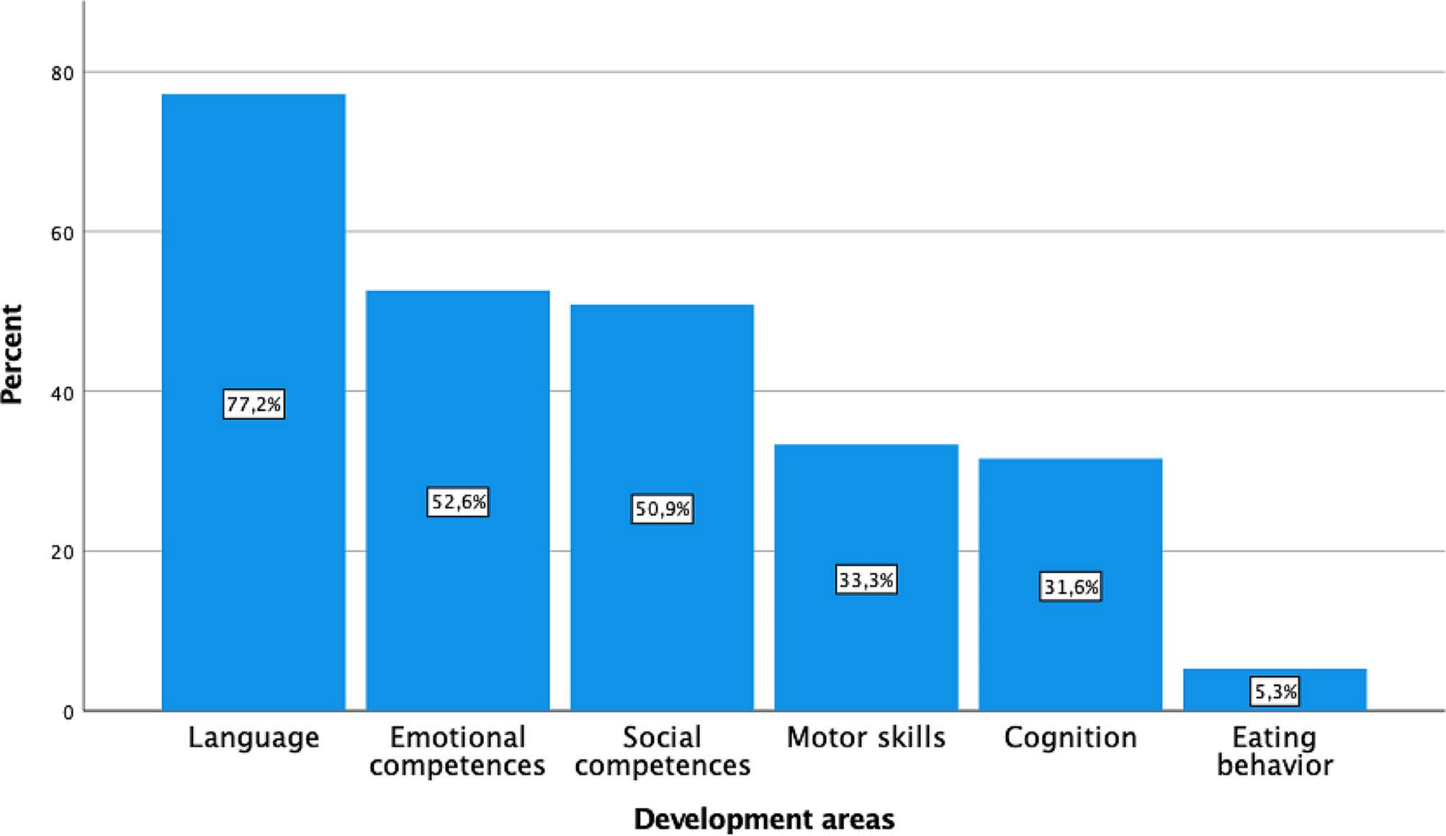
Developmental domains of concern for parents

In the survey, parents reported that they were concerned about the child’s development on average when the child was 1.8 years old (standard deviation, SD: 0.64, range: 2.75). Impaired language development was the most common cause of parental concern (77.2%, *n* = 44), followed by challenges in emotional (52.6%, *n* = 30) and social competences (50.9%, *n* = 29) (Fig. 1). Parents consulted a professional when their child had reached an average age of 2.7 years (SD: 0.8, range: 4.5) due to concerns about their child’s development. The pediatrician was the most commonly consulted professional (77.2%, *n* = 44). The ASD diagnosis was made at an average age of 3.7 years (SD: 0.8, range: 3), with a median waiting time of 3.0 months for the diagnostic workup (range: 11).

During the qualitative interviews, parents identified the length of the assessment process as the most significant challenge. Some 65% of the participating parents reported facing long waiting times and multiple appointments before receiving a diagnosis for their child. They described the assessment process as difficult and expressed a desire for a diagnosis to explain their child’s challenging behavior. Some 60% of the parents expressed the need for closer follow-up and more support in some way or another after receiving the diagnosis. During the interviews, some parents reported having requested a follow-up appointment at the location where the diagnostic workup was performed a few months after the diagnosis.

### Therapies

Our survey showed that at the time of it was conducted, children most frequently attended speech therapy (78.8%, *n* = 41), followed by occupational therapy (63.5%, *n* = 33) and special education (48.1%, *n* = 25). Before beginning kindergarten, all children received early special needs education (100%, *n* = 57), and 80.7% (*n* = 46) also attended speech therapy. Table 2 provides detailed information on the intensity, duration, and distance of early special needs education and speech therapy. About a quarter of children (24.6%, *n* = 14) participated in specific intensive intervention programs for ASD.

**Table 2:** Intensity, duration, distance of early special education and speech therapy

	Early special education [*n* = 57]		Speech therapy [*n* = 46]	
	n	%	n	%
**Intensity**				
0.5–1 h	21	36.8	25	54.4
1.5 h	3	5.3	2	4.3
2 h	24	42.1	18	39.1
2.5 h	2	3.5	0	0.0
> 2.5 h	7	12.3	1	2.2
Mean +/- SD [h/week]	1.8 +/- 0.8		1.5 +/- 0.6	
Minimum [h/week]	1		0.5	
Maximum [h/week]	5		4	
**Duration**				
0–6 months	2	3.5	7	15.2
7–12 months	20	35.1	18	39.1
13–18 months	7	12.3	5	10.9
19–24 months	19	33.3	10	21.7
> 24 months	9	15.8	6	13.0
Mean +/- SD [months]	20.2 +/- 10.6		17.9 +/- 13.2	
Minimum [months]	6		2	
Maximum [months]	60		60	
**Distance**				
0 km (at home)	31	54.4	1	2.2
0.1–5 km	14	24.6	28	60.9
5.1–10 km	7	12.3	10	21.7
10.1–15 km	4	7.0	1	2.2
> 15 km	1	1.8	1	2.2
Mean +/- SD [km]	2.75 +/- 4.9		5.0 +/- 4.9	
Minimum [km]	0		0	
Maximum [km]	23		27	

During the interviews, four parents noted that the therapies were logistically challenging due to their distance and the need to care for another child. Three parents reported difficulty in obtaining approval for sufficient therapy hours to be granted by the USNE. And 44% of the parents expressed a desire for more therapy sessions. A higher intensity of speech therapy was frequently mentioned as a desired improvement. In the survey, 16 parents expressed a desire for financial support for therapy costs. Half of these parents wanted the payment of intensive intervention programs. Additionally, four parents expressed a wish for more professional support and accompaniment.

### Everyday life experiences

**Fig. 2:**
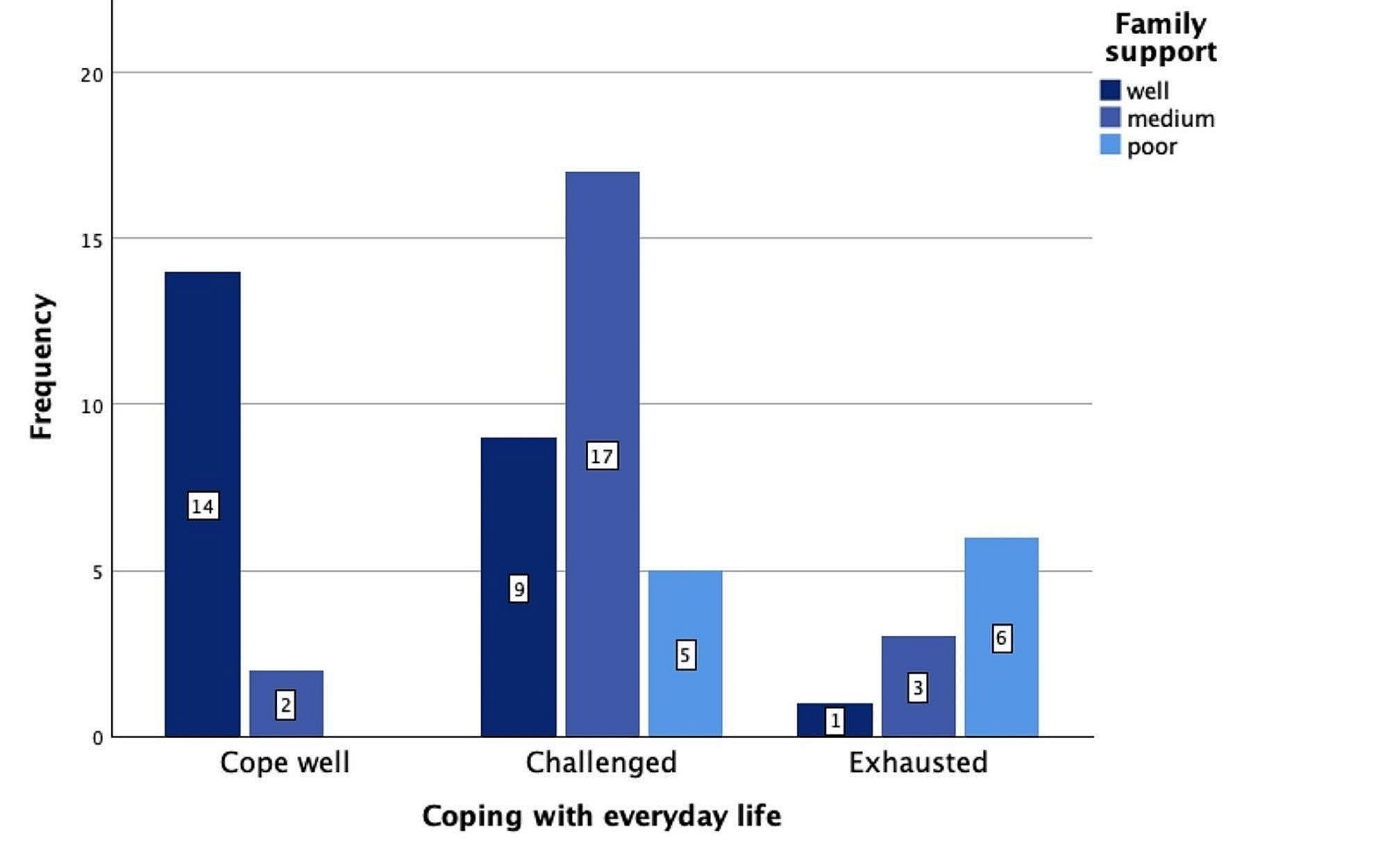
Association between Coping with Everyday Life Management and the Quality of Family Support

In the survey, we asked the parents how they cope with their daily lives on a 3-point rating scale (I manage my everyday life well, I feel challenged, I feel exhausted). In response, 28.1% (*n* = 16) stated that they manage their everyday lives well, 54.4% (*n* = 31) answered they felt challenged, and 17.5% (*n* = 10) felt exhausted. On a 3-point rating scale (good, moderate and poorly), 42.1% (*n* = 24) of the parents rated family support as good, 38.6% (*n* = 22) as moderate, and 19.3% (*n* = 11) as poor. Families who reported feeling exhausted also reported poor family support more frequently (*n* = 6) than families who reported successful everyday coping (*n* = 0), whereas families who reported successful everyday coping were more likely to report having good family support (*n* = 14) (see Fig. 2). Just over half of the parents (52.6%, *n* = 30) reported incurring additional expenses due to their child’s special needs. The expenses primarily arose from therapeutic interventions (60.0%, *n* = 18) and educational interventions (33.3%, *n* = 10). Over half of the families (54.4%, *n* = 31) had to reduce (24.6%, *n* = 14) or quit (29.8%, *n* = 17) their jobs due to the increased need for childcare.

In the qualitative interviews, nearly all of the parents reported an impact on the family’s daily life. “A child with ASD changes the life of a family radically. You are faced with challenges when you still don’t know how to handle them” (B09, pos. 156). The child on the autistic spectrum was, for example, described as the center of attention: “Of course, we arrange our whole lives, especially mine, around him” (B09, pos. 112). In addition, family activities were described as challenging. One mother reported that she had lost some friendships: “The nature of our son has deprived us of some friendships. I don’t know, people don’t handle it so well when a child is a little different” (B10, pos. 54). Some 40% of the parents interviewed reported the impact of the diagnosis on their well-being. They mentioned that both the diagnosis and the child’s behavior could sometimes cause stress. One mother told of physical symptoms triggered by this stress. However, the parents also mentioned positive effects of their child’s neurodivergent characteristics. Two mothers noted that their child’s special needs had made them personally more mature. One mother said that she often enjoyed the positive things about her child: “I can be very happy about these positive things that I would not experience with another child” (B10, pos. 70). More support in everyday life, for example a specialist case manager, was mentioned as a wish by more than half of the parents. Likewise, the wish for financial support was expressed by a third of the parents. One example was better payment in the sense of higher sums for interventions and support services. Another need expressed by six parents was for flexible jobs that are compatible with caring for a child with special needs.

## Discussion

Our study aimed to examine the perspectives of parents of children on the autism spectrum regarding the assessment process and provision of specific therapies and support in the Canton of Zurich, Switzerland. Parents emphasized the importance of early support that is tailored to their needs, even before a diagnosis is made. Despite a care system that is considered relatively good in general, parents expressed a desire for more assistance in multiple domains, including mastering challenges in everyday life and obtaining financial support.

On average, parents in our study were already concerned about the children’s development when the child was 1.8 years old - most commonly due to delayed language development. This is in line with results from other studies [26–28]. However, one of the most frequently mentioned worries was the duration of the diagnostic process, which was described as a time of uncertainty and unmet needs. Crane et al. (2018) also found that lack of support after diagnosis was particularly difficult for families as they try to understand the diagnosis and its implications. Those authors concluded that it is important to provide support for families during this time [29]. Our results also indicate that counseling and support should be provided promptly when parents express the need for it rather than only after the diagnosis. Therefore, it seems crucial for pediatricians not only to initiate the diagnostic process but also to evaluate each family’s support requirements regardless of the diagnosis. If necessary, pediatricians should consider initiating support, such as early special needs education, to assist and accompany families at an early stage. It therefore seems advisable to explicitly ask parents about their needs, to inform them about the various options available, and to involve them in joint decision-making at an early stage, as suggested by Locke et al. [21]. In our cohort, pediatricians were usually the professionals consulted first, and they played a central role in offering support to families. Therefore, it seems crucial that pediatricians are aware of the early autistic symptoms and know where to refer families for support. A recent survey among pediatricians in the Canton of Zurich revealed that most of them feel confident in accompanying families with children on the autism spectrum appropriately. However, they also reported feeling less confident in their knowledge about children on the autistic spectrum, which highlights the importance of providing more opportunities for primary care pediatricians to receive training and coaching on neurodiversity [30].

All children included in our study were enrolled for early special needs education, and most of them also underwent speech therapy. At school age, speech therapy was the most common therapy, followed by occupational therapy. Only about 25% of the children underwent an intensive intervention program for children on the autism spectrum. However, some of the other parents also expressed a desire for such therapy, which may indicate that demand is higher than supply. Additionally, these therapies are both time consuming and costly, which could suggest that the lack of therapy places available is not the only factor. Currently, extensive research is being conducted into a variety of therapeutic approaches. Sandbank et al. (2020) recommend that professionals caring for children on the autism spectrum inform families about the range of interventions available. Clinicians should make individualized recommendations to serve the child’s perceived needs and the family’s priorities [31]. This is supported by our study, in which many participating parents also expressed a need for more comprehensive support after diagnosis, including a follow-up appointment at the place of assessment.

Specialists in early special needs education play a crucial role in supporting and advising parents of neurodivergent children. In this setting, the focus is most often on the family, and early special needs educators provide parents with support on all aspects of everyday life. The importance of family involvement in early special needs education, its close adaptation to the family and the increasing interest of early special needs educators in further training in engaging with autism spectrum development indicate that early special needs education plays an increasingly vital role in supporting families with a child on the autism spectrum. Our findings reinforce the importance of providing parents with early special needs education support as soon as the need becomes evident, whether or not a diagnosis has been confirmed [32]. In this context, the relevance of parents’ collaboration with providers, as described by Moodie-Dyer et al., also appears to play an important role [20].

A significant number of parents reported feeling overwhelmed in their daily lives in our study, with 17.5% expressing exhaustion. These statements suggest that many parents of children on the autism spectrum experience significant subjective challenges and stress due to the level of care required for their child. Similar findings have been reported in other qualitative studies [11, 33]. Support from a social network seems to be important for coping with everyday life [34]. Our results also suggested that families with good family support were more likely to report good daily well-being. Our findings also support conclusions by Picardi et al. (2018), who stated that less social support was associated with higher parental stress [13], and by Kapp et al. (2011), who described how social support was a resilience-promoting factor in families with a child on the autistic spectrum [35].

One central issue for participants in our study was the financial challenge. Every second family stated that they faced additional costs. In addition, in more than half of the families, at least one parent had to reduce or even quit their job due to the child’s care needs. ASD has already been reported to be associated with a financial burden [11, 36, 37]. A study in the US (2007) concluded that this financial burden was not only present during childhood but persisted into adulthood due, for example, to caregiving in adulthood and potentially lower productivity of individuals on the autistic spectrum and their parents’ reduced earning capacity [38]. It is thus not surprising that many parents expressed their need for financial support. Therefore, it is important to create suitable financial conditions for individuals on the autism spectrum development at any age. Improved financial support, for instance from the insurance system, could contribute to achieving this goal. A study by Derguy et al. suggests that parents should also be supported by professionals who can inform them about the financial support to which they are entitled. [39].

Parents expressed a need for more support in multiple domains. The need for support, such as from professionals and other caregivers, was mentioned most frequently. Derguy at al. (2015) proposed that support for parents of children on the autism spectrum should cover all aspects of parenting [39]. Similar to previous findings, our results also suggest that the individual support needs of parents of children on the autism spectrum mean that different types of support are needed for different groups of parents, which presents an additional challenge to the system [40, 41]. Besides expressing the need for support, parents also emphasized the great value of living with their neurodivergent child and how they themselves can grow and mature in this relationship.

### Implications for practice

Our study contributes to the discourse about improving care for children on the autistic spectrum and their parents in several areas. Firstly, our findings add to the knowledge on the experiences of parents of children on the autism spectrum at different stages of life and the various needs they express. Thus, the results of this study could help to increase awareness of the different needs of different families with a child on the autistic spectrum and improve the coordination of support. Furthermore, we were able to confirm that family needs are highly individual and vary across life stages. Pediatricians and early special needs educators play vital roles in coordinating and supporting families, particularly in preschool settings. Our results may also prompt improvements in diagnostic workup and intervention procedures by enhancing accessibility and coordination. All professionals who care for and accompany children on the autism spectrum should have ample opportunities for further education about this condition.

### Study limitations and directions for further research

Our study has several limitations. Firstly, data was only collected at one point in time, which restricts the understanding of changes and progress in parents’ experiences and needs over time. Additionally, this is a single-center study with a limited sample size, which may limit generalizability. However, given the low prevalence of ASD compared to other conditions, we nevertheless consider our results to also be relevant for other contexts, especially in less well-resourced regions and countries. Another weakness is the self-selection of parents, because parents that choose to participate may be more active in seeking support and expressing their needs. Furthermore, the study only included parents with children registered at the USNEs. However, we know from clinical experience that children on the autism spectrum are mostly identified before kindergarten entry and therefore are registered at the USNE so that the number of children, who are not assigned to the USNE should be small. Furthermore, more than half of the original population of 147 consisted of families whose native language was not German [42]. Because we sent the questionnaire only in German, English, or Albanian and the interview was conducted only in German, our results might underrepresent the experiences of non-German-speaking families. Another limitation is the lack of comparison with a control group of neurotypical children or children with other developmental challenges. Finally, more detailed sample characterization would have been desirable. Nevertheless, the results of this study provide a good overview of the care situation of families with a child on the autism spectrum in the Canton of Zurich, which also allows conclusions to be drawn that can be applied to other regions and countries.

## Conclusions

In most cases, parents of children on the autism spectrum first consult their pediatrician if they are concerned about the development of their child, which is often long before the diagnosis is established. The families described very different needs for various types of support, and these needs arose early. Pediatricians therefore play a central role in assisting the families and guiding them through the system of medical assessments, the diagnostic process, early interventions, and financial support. Counseling parents about support should start early, whether or not the diagnosis has been established. Early childhood special needs educators play an increasingly important role in supporting families with a neurodivergent child in Switzerland. Their approach, involving the whole family, seems to meet one of the parents’ priority needs. Early family support also seems to have an influence on the parents’ ability to manage everyday life, which highlights the positive effect of interventions by early childhood special needs educators. Financial challenges are an issue for many parents of children on the autism spectrum, in many cases even limiting access to special intensive intervention programs. Low-threshold financial counseling should therefore be available to the families. Despite the sometimes substantial challenges and the resultant need for support, many parents emphasize the benefit that life with a neurodivergent child offers and how they have grown as a result.

## Electronic supplementary material

Below is the link to the electronic supplementary material.

Supplementary Material 1

Supplementary Material 2

## Data Availability

The data that support the findings of this study are not openly available due to reasons of sensitivity and are available from the corresponding author upon reasonable request. Data are located in controlled access data storage at the University Children`s Hospital Zurich.

## Abbreviations

ASD: Autism spectrum development
ADOS: Autism diagnostic observation schedule
SD: Standard deviation
USNEs: Units for special needs education
