# Supplementary material for: Autism spectrum: parents’ perspectives reflecting the different needs of different families
Source: BMC Pediatr. 2024 Jul 9;24:439. doi: 10.1186/s12887-024-04912-x (PMC11232266; doi:10.1186/s12887-024-04912-x)
Supplement: Supplementary file 1 — Supplementary Material 1 [file 12887_2024_4912_MOESM1_ESM.docx]

**Supplementary Material**

**Questionnaire (English translation)**

**Children with Autism Spectrum Disorder in the canton of Zurich**

Dear Parents,

We are currently conducting a scientific study at the Children's Hospital in Zurich in which we are studying the living situations of families with children who have autism spectrum disorder (ASD). This is why we are inviting families whose child was diagnosed with ASD and also initiated early remedial education between 2014 and 2017 to participate in the study.

We would like to ask you, being the parents, **how you are coping with your current situation as a family with a child with ASD and, in retrospect, how you have coped with the development, the clarification process and the therapies that your child has had to undergo.** It will be about your experiences and needs with regard to your child's diagnosis and the therapies involved. The focus will be on your child who has been diagnosed with ASD as well as on you, being the child's mother / father / caregiver. The object of the survey is to better understand the needs of the parents in order to further improve the support provided to children with ASD. Completing the questionnaire (Parts A – C) should take you **15-20 minutes**.

We would be very grateful if you would participate in the survey!

All of the information that you provide in the following questionnaire will be **treated as strictly confidential and evaluated in encrypted form**, so that someone who is not involved in the research project cannot draw any conclusions about your child or yourself. We would like to thank you in advance for sharing your assessments candidly and openly. This means that you will be making a significant contribution to ensuring that the support provided to children with ASD can be further improved in the future. Ms. Nadja Schneider will be happy to help you (Tel.: 044 266 7146 or e-mail her at: nadja.schneider@kispi.uzh.ch) if you have any questions about the questionnaire or the study.

Please send the **completed questionnaire and the signed consent form** back to us in the enclosed envelope by ***28.02.2021***.

Thank you for your cooperation!

**List of abbreviations used in the questionnaire**

| FIAS | Frühintervention bei autistischen Störungen (Basel) |
| --- | --- |
| FIVTI | Frühe intensive verhaltenstherapeutische Intervention (Zurich) |
| ABA | Applied Behaviour Analysis |
| DIR | Developmental Individual Differences Relationship |
| RDI | Relationship Development Intervention |

| ID: | Child’s surname | Child's first name |
| --- | --- | --- |
|  |  |  |

**Part A: Current situation**

Part A of the questionnaire asks about your child's **current life situation (in relation to the last 3 months)**. Cross the appropriate answers to the corresponding questions (multiple answers are possible).

1. Details about your child's development

Actual age: _______________ years, ______________ months

In which stage of your child's development did he/she start to fall behind that of other children? Please indicate how pronounced the delayed development was.

|  | No delay | Slight delay | Noticeable delay | Severe delay |
| --- | --- | --- | --- | --- |
| Speech |  |  |  |  |
| Movement / Motor skills |  |  |  |  |
| Thinking / Cognition |  |  |  |  |
| Social skills |  |  |  |  |
| Emotional development |  |  |  |  |

| Comment: |  |
| --- | --- |

1. Details about your child's therapies

Which therapy types is your child currently using? Please state the number of hours per week for therapy types being used. (Multiple answers are possible)

| Therapy type(s) |  | Often |
| --- | --- | --- |
| None |  |  |
| Remedial education |  | Hours per week |
| Speech therapy |  | Hours per week |
| Autism-specific intervention therapy (FIAS, FIVTI) | EIAD  EIBI | Hours per week |
| Autism-specific interventions (ABA, DIR, RDI) | ABA  DIR  RDI | Hours per week |
| Psychotherapy |  | Hours per week |
| Occupational therapy |  | Hours per week |
| Other: ________________________ |  | Hours per week |

| Comment: |  |
| --- | --- |

How far is it between your place of residence and where you have to go for the type of therapy needed? Please state the number of kilometres.

| Therapy | Number of kilometres |
| --- | --- |
| Remedial education |  |
| Speech therapy |  |
| Autism-specific intervention therapy (FIAS, FIVTI) |  |
| Autism-specific interventions (ABA, DIR, RDI) |  |
| Psychotherapy |  |
| Occupational therapy |  |
| Other: _____________________________ |  |

How do you rate your satisfaction with your child's therapeutic measures?
0 = dissatisfied, 10 = very satisfied (please mark with a cross)

| 0 | 1 | 2 | 3 | 4 | 5 | 6 | 7 | 8 | 9 | 10 |
| --- | --- | --- | --- | --- | --- | --- | --- | --- | --- | --- |
|  |  |  |  |  |  |  |  |  |  |  |

What else would you like to see? ______________________________________________________________________________________________________________________________________________________________________________________________________________________________

| Comment: |  |
| --- | --- |

1. Details about your child's education

Current type of school or kindergarten:

|  | Type of school | Detailed description | |
| --- | --- | --- | --- |
|  | Kindergarten | Entry date: ______________________ (month/year) | |
|  |  |  | Normal kindergarten without special educational measures |
|  |  |  | Normal kindergarten with special educational measures |
|  |  |  | Small class |
|  |  |  | Remedial education kindergarten |
|  |  |  | Speech therapy school |
|  |  |  | Other: ________________________________________ |
|  | School | Entry date: ______________________ (month/year)  Class: ____________________ | |
|  |  |  | Normal school without special educational measures |
|  |  |  | Normal school with special educational measures |
|  |  |  | Small class |
|  |  |  | Remedial education school |
|  |  |  | Speech therapy school |
|  |  |  | Other: ________________________________________ |

| Comment: |  |
| --- | --- |

How far is it between your place of residence and the type of school or kindergarten your child is currently attending? Please state the number of kilometres.

Number of kilometres: ________________________________________________________

How do you rate your satisfaction with your child's education?
0 = dissatisfied, 10 = very satisfied (please mark with a cross)

| 0 | 1 | 2 | 3 | 4 | 5 | 6 | 7 | 8 | 9 | 10 |
| --- | --- | --- | --- | --- | --- | --- | --- | --- | --- | --- |
|  |  |  |  |  |  |  |  |  |  |  |

What else would you like to see?

______________________________________________________________________________________________________________________________________________________________________________________________________________________________

| Comment: |  |
| --- | --- |

1. Details about your child's leisure time (multiple answers are possible)

Who did your child have contact with during her/his leisure time before the COVID-19 pandemic? Please state the frequency.

|  | Contact | Often | |
| --- | --- | --- | --- |
|  | Parents |  | Seldom |
|  |  |  | Now and then |
|  |  |  | Often |
|  | Brothers and sisters |  | Seldom |
|  |  |  | Now and then |
|  |  |  | Often |
|  | Other relatives (uncle(s), aunt(s), cousin(s), etc.) |  | Seldom |
|  |  |  | Now and then |
|  |  |  | Often |
|  | Classmates |  | Seldom |
|  |  |  | Now and then |
|  |  |  | Often |
|  | Friends  Number: ______________________ |  | Seldom |
|  |  |  | Now and then |
|  |  |  | Often |
|  | Club contacts  Which: ______________________ |  | Seldom |
|  |  |  | Now and then |
|  |  |  | Often |

|  | Neighbours |  | Seldom |
| --- | --- | --- | --- |
|  |  |  | Now and then |
|  |  |  | Often |
|  | Other:  __________________________ |  | Seldom |
|  |  |  | Now and then |
|  |  |  | Often |

What does your child do in her/his leisure time? Please refer to the time before the COVID-19 pandemic as well here. Please state the frequency.

|  | Leisure activities | Often |
| --- | --- | --- |
|  | Family activities:  Which:  __________________________ __________________________ | Hours per day: |
|  | TV | Hours per day: |
|  | Computer | Hours per day: |
|  | Sporting activities | Hours per week: |
|  | Meeting friends | Hours per week: |
|  | Reading | Hours per day: |
|  | Creative activities  Which: __________________________ __________________________ | Hours per week: |
|  | Other: ________________________ | Hours per day: |

If you would have liked or would like to have additional leisure activities for your child, what would they be?

______________________________________________________________________________________________________________________________________________________________________________________________________________________________

How do you rate your satisfaction with your child's leisure time activities?
0 = dissatisfied, 10 = very satisfied (please mark with a cross)

| 0 | 1 | 2 | 3 | 4 | 5 | 6 | 7 | 8 | 9 | 10 |
| --- | --- | --- | --- | --- | --- | --- | --- | --- | --- | --- |
|  |  |  |  |  |  |  |  |  |  |  |

What else would you like to see?

______________________________________________________________________________________________________________________________________________________________________________________________________________________________

| Comment: |  |
| --- | --- |

1. Details about your living situation / your everyday life

How do you cope with everyday tasks as a parent of an autistic child?

I cope well with everyday life

I feel challenged

I feel exhausted

How do you feel with regard to being supported in a family environment?

| Good | So-so | Poorly |
| --- | --- | --- |
|  |  |  |

How understandingly did those around you react to your child's diagnosis?

|  | Not very understanding |
| --- | --- |
|  | Their understanding was so-so |
|  | Very understanding |

What or who are the important resources or people that support you?

______________________________________________________________________________________________________________________________________________________________________________________________________________________________

| Comment: |  |
| --- | --- |

Do you have contact with other families with an autistic child?
If yes, how often and in what form do these contacts take place?

|  | No | | | |
| --- | --- | --- | --- | --- |
|  | Yes | **Often** | | **Type** |
|  |  |  | Seldom |  |
|  |  |  | Now and then |  |
|  |  |  | Often |  |

How do you rate your satisfaction with your role as a parent?
0 = dissatisfied, 10 = very satisfied (please mark with a cross)

| 0 | 1 | 2 | 3 | 4 | 5 | 6 | 7 | 8 | 9 | 10 |
| --- | --- | --- | --- | --- | --- | --- | --- | --- | --- | --- |
|  |  |  |  |  |  |  |  |  |  |  |

What else would you like to see?

______________________________________________________________________________________________________________________________________________________________________________________________________________________________

| Comment: |  |
| --- | --- |

1. Details about legal and financial issues

Has a birth defect 405 (GG 405) been registered with the DI (Disability Insurance)?
If yes, did the DI accept your child's GG 405?

|  | No | | | |
| --- | --- | --- | --- | --- |
|  | Yes | **Was the GG 405 accepted by the DI?** |  | No |
|  |  |  |  | Yes |

Has an application for helplessness allowance been submitted to the DI?
If yes, was it approved?

|  | No | | | |  |
| --- | --- | --- | --- | --- | --- |
|  | Yes | **Application approved?** |  | No | |
|  |  |  |  | Yes | |

If the application for helplessness allowance was approved, which level was approved? From when did the financial allowance start?

|  | Helplessness allowance level | |
| --- | --- | --- |
|  | Low level | |
|  | Mid-level | |
|  | Full level | |
|  | How old was your child when the allowance started? |  |

| Comment: |  |
| --- | --- |

Have you incurred any additional costs due to your child's need for support that you have had to finance privately?
If yes, what for?

|  | No |
| --- | --- |
|  | Yes, for the following therapeutical measures: ________________________________________________________________ |
|  | Yes, for the following educational measures: ________________________________________________________________ |
|  | Yes, for visiting the following facilities / institutions: ________________________________________________________________ |
|  | Yes, for the following aids: ________________________________________________________________ |
|  | Other: ________________________________________________________________ |

In your opinion, which costs that have not yet been covered by DI or health insurance should be covered?

______________________________________________________________________________________________________________________________________________________________________________________________________________________________

Have you received support from a social counselling specialist with regard to financial issues?
If yes, what type of support was provided?

|  | No | |
| --- | --- | --- |
|  | Yes | **Type** |
|  |  |  |

Have you had to reduce or even give up your work / career due to your child's increased support needs?
If yes, how severe was the resulting financial loss?

|  | No | | |
| --- | --- | --- | --- |
|  | Yes, I have had to reduce my time at work | **Level of financial loss** | |
|  |  |  | Little |
|  | Yes, I had to give up my job |  | So-so |
|  |  |  | Considerable |

How do you rate your satisfaction in relation to your financial situation?
0 = dissatisfied, 10 = very satisfied (please mark with a cross)

| 0 | 1 | 2 | 3 | 4 | 5 | 6 | 7 | 8 | 9 | 10 |
| --- | --- | --- | --- | --- | --- | --- | --- | --- | --- | --- |
|  |  |  |  |  |  |  |  |  |  |  |

What else would you like to see?

______________________________________________________________________________________________________________________________________________________________________________________________________________________________

| Comment: |  |
| --- | --- |

**Part B: Review**

Part B of the questionnaire asks you to review **your child's life situation during the past (refers to the period > 1 year ago)**. Cross the appropriate answers to the corresponding questions (multiple answers are possible).

1. Details about your child's development

Age when the first signs of delayed development started to appear:

__________ years __________ months

In which part of your child's development did abnormalities first appear?

Speech

Movement / Motor skills

Thinking / Cognition

Social skills

Emotional skills

Who was the first to notice this delayed development?

Mother

Father

One of our relatives

A friend

A specialist: _______________________________________________________
 Please state their professional category

| Comment: |  |
| --- | --- |

1. Details about your child's clarification process

Age when diagnosed: __________ years __________ months

Who did you make initial contact with about the developmental delays that were noticed?

Paediatrician

Family doctor

Other: ____________________________________________________________

How old was your child when you first consulted a specialist because of the delays that you noticed? __________ years __________ months

How long was the wait before your child received a clarification appointment? _______________ months

Who made the diagnosis?

Paediatrician

Developmental paediatrics at the University Children's Hospital in Zurich

Developmental paediatrics at the Cantonal Hospital in Winterthur

Resident specialist in developmental paediatrics

Child and Adolescent Psychiatry, Zurich

Other: _____________________________________________________________

Who advised you and informed you after the diagnosis?

____________________________________________________________________________________________________________________________________________________

How do you rate the advice and information that you were given after your child's diagnosis?
0 = not informed / advised at all, 10 = very well informed / advised (please mark with a cross)

| 0 | 1 | 2 | 3 | 4 | 5 | 6 | 7 | 8 | 9 | 10 |
| --- | --- | --- | --- | --- | --- | --- | --- | --- | --- | --- |
|  |  |  |  |  |  |  |  |  |  |  |

What other clarifications were made?

None

Genetics

Metabolic screening

Neurology

Other: _____________________________________________________________

How far is it between your place of residence and where you had to go for the clarification?

Number of kilometres: _________________________

How do you rate your satisfaction in relation to your child's clarification process?
0 = dissatisfied, 10 = very satisfied (please mark with a cross)

| 0 | 1 | 2 | 3 | 4 | 5 | 6 | 7 | 8 | 9 | 10 |
| --- | --- | --- | --- | --- | --- | --- | --- | --- | --- | --- |
|  |  |  |  |  |  |  |  |  |  |  |

What else would you like to see?

____________________________________________________________________________________________________________________________________________________

| Comment: |  |
| --- | --- |

1. Details about your child's therapies

Which special educational measures did your child receive in the past? Please state the frequency and duration of the support for the measures you have crossed.

| Measure |  | Often | Duration |
| --- | --- | --- | --- |
| None |  |  |  |
| Early remedial education |  | Hours per week | Months |
| Speech therapy |  | Hours per week | Months |

What autism-specific support did your child receive? Please state the frequency and duration of the support for the measures you have crossed.

| Autism-specific support |  | Often | Duration |
| --- | --- | --- | --- |
| None |  |  |  |
| ABA (Applied Behavioural Analysis) |  | Hours per week | Months |
| RDI (Relationship Development Intervention) |  | Hours per week | Months |
| Floortime (DIR = Developmental Individual Differences Relationship) |  | Hours per week | Months |
| FIVTI (Zurich) |  | Hours per week | Months |
| FIAS (Basel) |  | Hours per week | Months |
| Other: ___________________ |  | Hours per week | Months |

If you would have liked or would like to have additional offers of help for your child, what would they be?

______________________________________________________________________________________________________________________________________________________________________________________________________________________________

Who advised and informed you about the therapeutic measures?

____________________________________________________________________________________________________________________________________________________

How do you rate the advice and information that you were given in relation to your child's therapeutic measures?
0 = not informed / advised at all, 10 = very well informed / advised (please mark with a cross)

| 0 | 1 | 2 | 3 | 4 | 5 | 6 | 7 | 8 | 9 | 10 |
| --- | --- | --- | --- | --- | --- | --- | --- | --- | --- | --- |
|  |  |  |  |  |  |  |  |  |  |  |

How far was it between your place of residence and where you had to go for the therapy? Please state the number of kilometres.

| Therapy | Number of kilometres |
| --- | --- |
| Early remedial education |  |
| Speech therapy |  |
| Autism-specific support |  |

| Comment: |  |
| --- | --- |

1. Details about your child's education

Which types of school or kindergarten has your child already attended? (Multiple answers are possible)

|  | Type of school | Detailed description | |
| --- | --- | --- | --- |
|  | Kindergarten | Entry date: ______________________ (month/year) | |
|  |  |  | Normal kindergarten without special educational measures |
|  |  |  | Normal kindergarten with special educational measures |
|  |  |  | Small class |
|  |  |  | Remedial education kindergarten |
|  |  |  | Speech therapy school |
|  |  |  | Other: ________________________________________ |
|  | School | Entry date: ______________________ (month/year)  Class: __________________ | |
|  |  |  | Normal school without special educational measures |
|  |  |  | Normal school with special educational measures |
|  |  |  | Small class |
|  |  |  | Remedial education school |
|  |  |  | Speech therapy school |
|  |  |  | Other: ________________________________________ |

How far is it between your place of residence and the type of kindergarten / school that your child previously went to? Please state the number of kilometres.

Number of kilometres: __________________________

Did you receive advice about the best possible type of school for your child? If yes, by whom and in what form?

|  | No | | |
| --- | --- | --- | --- |
|  | Yes | **Professional category of the person who advised you** | **Type** |
|  |  |  |  |

How do you rate the advice and information that you were given about your child's optimum education?
0 = not informed / advised at all, 10 = very well informed / advised (please mark with a cross)

| 0 | 1 | 2 | 3 | 4 | 5 | 6 | 7 | 8 | 9 | 10 |
| --- | --- | --- | --- | --- | --- | --- | --- | --- | --- | --- |
|  |  |  |  |  |  |  |  |  |  |  |

| Comment: |  |
| --- | --- |

**Part C: Statistical data**

The following data provided by you will enable us to evaluate the results in a targeted manner. This is important as it will enable us to make accurate conclusions afterwards.

1. Details about your child who has been diagnosed with autism

Gender: Male Female

Age: __________ years __________ months

Which autism diagnosis was your child given? _________________________________________________________________

Other medical diagnoses: ____________________________________________

1. Details about the child's parents

|  | **Mother** | | **Father** | |
| --- | --- | --- | --- | --- |
| Age |  | |  | |
| First language |  | |  | |
| Country of origin |  | |  | |
| Lived in Switzerland since |  | |  | |
| Profession and workload (in %) |  | |  | |
| Highest school-leaving qualification |  | Compulsory schooling (prim., sec.) |  | Compulsory schooling (prim., sec.) |
|  |  | Apprenticeship, pre-vocational school |  | Apprenticeship, pre-vocational school |
|  |  | Vocational training |  | Vocational training |
|  |  | Secondary school / Grammar school |  | Secondary school / Grammar school |
|  |  | Higher technical and vocational education |  | Higher technical and vocational education |
|  |  | Technical college |  | Technical college |
|  |  | University / Swiss Federal Institute of Technology |  | University / Swiss Federal Institute of Technology |
|  |  | None |  | None |

1. Details about your family

Residence: ____________________________________________________________

How many people live in your household?

______ adults (16 or older) ______ children

How many rooms does your apartment have (do not include the bathroom and kitchen)?

________________________________

I am the mother / father of the child

| Comment: |  |
| --- | --- |

Finally, we would like to ask you if you would, in principle, be willing to participate in an interview. If yes, please let us know when we can best reach you by phone to arrange an appointment for the interview.

|  | No | | |
| --- | --- | --- | --- |
|  | Yes | Best time to contact me by telephone: (Please state the weekday and the time.) |  |

**Thank you for your valuable cooperation!**

**Please send the questionnaire and the signed consent form back to us.**

**Interview template**

**Gesprächsleitfaden für das Elterninterview**

Version: Interviews, 03. 12. 2020

| **Allgemeine Informationen zum Interview** |
| --- |

- Zum Einstieg des Gespräches wird die Mutter/der Vater willkommen geheissen und über Inhalte und Ziele des Interviews informiert.
- Nach allgemeinen Fragen zur Familie werden die **Hauptfragen A bis F** gestellt. Da es sich um ein halbstrukturiertes Interview handelt, besteht die Möglichkeit, die Fragen in einer anderen Reihenfolge zu besprechen, um die Antworten und Erzählungen situativ in das Gespräch aufnehmen und einen fliessenden Austausch gewährleisten zu können. Die Unterfragen werden nur gestellt, wenn die entsprechenden Themen nicht bereits von der Mutter/dem Vater angesprochen wurden.
- Im **Teil G** werden individuell formulierte Fragen gestellt – gemäss Auswertung des Fragebogens, welcher von der Mutter/dem Vater im Voraus schriftlich ausgefüllt wurde. So kann beispielweise gefragt werden, weshalb die Eltern beispielsweise so einschätzen wie angegeben, und wie sich dies auswirkt.
- Der **Schlussteil** beinhaltet einen offenen Rahmen für weitere Anmerkungen und Fragen sowie ein Dankeschön für die Teilnahme.
- Die kursiv geschriebenen Sätze werden gesprochen. Die blau hinterlegt Angaben sind Informationen aus dem Fragebogen.

| **Einstieg** |
| --- |

Joining

*Ich begrüsse Sie zu diesem Gespräch und möchte mich herzlich bedanken, dass Sie sich die Zeit nehmen, mit mir zu sprechen und einige Fragen zu beantworten.*

[Interviewerin stellt sich selbst und den Bezug zum Projekt kurz vor]

Ziel des Gespräches

*Im Rahmen meiner [Doktorarbeit] interessiert mich, wie Eltern von Kindern mit einer Autismus-Spektrum-Störung die aktuelle Situation als Familie einschätzen und wie Sie rückblickend die Entwicklung, den Abklärungsprozess und die therapeutischen Massnahmen Ihres Kindes erlebt haben. In unserem Gespräch heute wird es vor allem darum gehen, was Ihre Erfahrungen sowie Ihre Wünsche und Bedürfnisse im Zusammenhang mit der Autismus-Spektrum-Störung Ihres Kindes sind. Dabei gibt es kein richtig oder falsch – uns interessieren Ihre Erfahrung, so wie Sie es erlebt haben.*

Ablauf des Interviews

*Das Gespräch wird 60 bis 90 Minuten dauern. Zu den Fragen, die ich Ihnen stellen werde, dürfen Sie frei erzählen, was Ihnen in den Sinn kommt. Mich interessieren Ihre persönlichen Einschätzungen. Es gibt also kein richtig oder falsch. Sie müssen keine Fragen beantworten, welche Ihnen unangenehm sind oder Sie nicht beantworten möchten. Wenn Sie das Interview abbrechen möchten, dürfen Sie dies jederzeit tun. Zwischenfragen dürfen Sie jederzeit gerne stellen.*

Aufzeichnung und Anonymisierung

*Ich werde das Gespräch mit dem Diktiergerät aufnehmen. Die Aufnahme werde ich dann in Schrift übertragen. Dabei werde ich Ihren Namen und alle anderen erwähnten Namen und Bezeichnungen anonymisieren. Für die Auswertung wird dann nur der anonymisierte Text verwendet, die Audiodatei wird gelöscht.*

Inhalte des Interviews

*Das Interview wird folgende Themen haben:*

- *Fragen zur Familie*
- *A Entwicklungsverlauf des Kindes*
- *B Erleben des Abklärungsprozess*
- *C Erleben der therapeutischen Massnahmen*
- *D Erleben des Bereichs Schulbildung*
- *E Erleben der Freizeit des Kindes*
- *F Lebenssituation der Familie*
- *G Bezug zum Fragebogen*

*Haben Sie jetzt gerade noch Fragen? Dann würden wir mit dem Interview beginnen.*

[Einschalten des Aufnahmegeräts]

Fragen zur Familie

*Zu Beginn würde ich mich freuen, Ihre Familie etwas kennenzulernen.*

- *Wer gehört alles zu Ihrer Familie?* (inkl. Alter der Geschwister)
- *Wie teilen Sie als Eltern die Familienarbeit und die Erwerbsarbeit auf?*
- *Wie sind Ihre Kinder betreut?* (z.B. Grosseltern, Kindertagesstätte)
- *Seit wann leben Sie in der Schweiz? Welches ist Ihr Herkunftsland?*
- *Welche Sprachen sprechen Sie zuhause?*

| **Interview-Leitfaden** |
| --- |

**A Entwicklungsverlauf des Kindes**

***Wie hat sich Ihr Kind entwickelt?***

1. Entwicklung des Kindes vor der Diagnosestellung

- *Wie ging es Ihrem Kind damals?*
- *Was waren die ersten Auffälligkeiten, die Ihr Kind zeigte?*
- *Was haben Sie gemacht/wie haben Sie darauf reagiert?*

1. Entwicklung des Kindes nach der Diagnosestellung

- *Wie ging es Ihrem Kind damals?*
- *Welche Entwicklungsschritte hat Ihr Kind in dieser Zeit gemacht?*
- *Gab es auch Rückschritte in der Entwicklung ihres Kindes?*

1. Entwicklung des Kindes aktuell

- *Wie geht es Ihrem Kind heute?*
- *Wie geht es Ihnen als Eltern/Familie heute?*
- *Was bereitet Ihnen Freude? Was ist schwierig?*

**B Erleben des Abklärungsprozess**

***Ihr Kind erhielt im [Monat, Jahr] die Diagnose Autismus-Spektrum-Störung. Wenn Sie an die Zeit des Abklärungsprozesses zurückdenken: Wie haben Sie den Abklärungsprozess erlebt / in Erinnerung?***

1. Situation 🡪 Setting Abklärungsprozess: Wo, wie lang, wie viele Termine
2. Eigene Erinnerungen der Eltern

- *Die Zeit liegt bereits etwas zurück, was fällt Ihnen als Erstes ein, wenn Sie an den Abklärungsprozess denken?*
- *Wie ging es Ihnen als Eltern / Familie damals? Was war belastend in dieser Zeit, was unterstützend?*
- *Wie wurden sie bezüglich der Diagnose informiert und beraten? Von wem?*
- *Welche Themen/Anliegen waren Ihnen damals wichtig?*

1. Bewertungen der Eltern

- *Wie zufrieden waren sie im Allgemeinen mit dem Abklärungsprozess?*
- *Gibt es etwas, was Sie besonders gut in Erinnerung haben* (positive Momente / Situationen)*? Gibt es etwas, was Sie eher schlecht in Erinnerung haben* (negative Momente / Situationen)*?*
- *Was würden Sie sich anders wünschen? Was könnte man verbessern?*

**C Erleben der therapeutischen Massnahmen**

***Ihr Kind erhielt heilpädagogische Früherziehung sowie [weitere Massnahmen]. Wenn Sie an diese Zeit zurückdenken: Wie haben Sie die HFE sowie die anderen Massnahmen erlebt / in Erinnerung?***

1. Situation

- *Welche therapeutischen Massnahmen erhielt Ihr Kind?*
- Setting: Wie lange, wie häufig

1. Eigene Erinnerungen der Eltern

- *Die Zeit liegt bereits etwas zurück, was fällt Ihnen als Erstes ein, wenn Sie an die therapeutischen Massnahmen Ihres Kindes denken?*
- *Was fällt Ihnen als Erstes ein, wenn Sie an die HFE / Massnahmen denken?*
- *Wie ging es Ihnen als Eltern/Familie damals? Was war belastend in dieser Zeit, was unterstützend?*
- *Welche Themen/Anliegen waren Ihnen damals wichtig?*
- *Wie wurden Sie als Eltern bezüglich der Wahl der geeigneten therapeutischen Massnahmen unterstützt? Wer hat Sie über die verschiedenen therapeutischen Massnahmen informiert und beraten? Wurden Sie über autismusspezifische Interventionen informiert?*
- *Durch welche therapeutische Massnahme konnte Ihr Kind Ihres Erachtens Fortschritte machen? In welchen Bereichen? Wie haben Sie als Familie davon profitiert?*

1. Bewertungen der Eltern

- *Wie zufrieden sind Sie im Allgemeinen mit den therapeutischen Massnahmen?*
- *Gibt es etwas, was Sie besonders gut in Erinnerung haben* (positive Momente / Situationen)*? Gibt es etwas, was Sie eher schlecht in Erinnerung haben* (negative Momente / Situationen)*?*
- *Was sind für Sie als Eltern die wichtigsten Therapieziele?*
- *Welche Intensität der therapeutischen Massnahmen ist für Sie wünschenswert? Hätten Sie sich mehr/weniger gewünscht? Wie schätzen die Sie die Distanz zum Therapieort ein? Wie aufwändig war es für Sie einen Platz zu erhalten?*
- *Was würden Sie sich anders wünschen? Was könnte man verbessern?*

1. Aktuelle Situation

- *Welche therapeutischen Massnahmen erhält Ihr Kind aktuell?*
- *Setting Massnahmen: Wie lange, wie häufig*
- *Ist die aktuelle Situation in Bezug auf die therapeutischen Massnahmen für Sie zufriedenstellend?*
- *Was würden Sie sich anders wünschen? Was könnte man verbessern? Welche Bedürfnisse haben Sie in diesem Bereich?*

**D Erleben des Bereichs Bildung**

***Ihr Kind besucht aktuell die [Schule]. Wenn Sie an den Bildungsweg Ihres Kindes denken: Wie haben Sie dies erlebt / in Erinnerung?***

1. Situation

- *Welche Schul-/Kindergartenformen besucht/-e Ihr Kind?*

1. Eigene Erinnerungen der Eltern

- *Was fällt Ihnen als Erstes ein, wenn Sie an den Bildungsweg Ihres Kindes denken?*
- *Wie ging / geht es Ihnen als Eltern / Familie (damals)? Was war / ist belastend in dieser Zeit, was unterstützend?*
- *Welche Themen / Anliegen waren / sind Ihnen wichtig?*
- *Weshalb wurde dieses oder jenes Schul- / Kindergartensetting ausgewählt?*
- *Wie wurden Sie als Eltern in der Wahl der bestmöglichen Schul-/Kindergartenform unterstützt? Wer hat sie in diesem Bereich informiert und beraten?*

1. Bewertungen der Eltern

- *Wie zufrieden sind Sie im Allgemeinen mit der Schulsituation Ihres Kindes?*
- *Gibt es etwas, was Sie besonders gut in Erinnerung haben* (positive Momente / Situationen)*? Gibt es etwas, was Sie eher schlecht in Erinnerung haben* (negative Momente / Situationen)*?*
- *Was würden Sie sich anders wünschen? Was könnte man verbessern? Welche Bedürfnisse haben Sie in diesem Bereich?*

**E Erleben der Freizeit des Kindes**

***Ihr Kind macht in der Freizeit gerne [Freizeitgestaltung]. Wenn Sie an die Freizeitgestaltung Ihres Kindes denken: Wie erleben Sie dies?***

1. Situation

- *Wie sieht die Freizeitgestaltung Ihres Kindes aus?*

1. Eigene Erinnerungen der Eltern

- *Was fällt Ihnen als Erstes ein, wenn Sie an die Freizeitgestaltung Ihres Kindes denken?*
- *Wie geht es Ihnen als Eltern/Familie damit? Was ist aktuell belastend, was unterstützend?*
- *Welche Themen/Anliegen sind Ihnen wichtig?*
- *Welche Unterstützung erhalten Sie als Eltern / Familie heute? Wäre Bedarf an familienbegleitenden Massnahmen da (gewesen)? Warum, warum nicht?*

1. Bewertungen der Eltern

- *Wie zufrieden sind Sie im Allgemeinen mit der Freizeitgestaltung Ihres Kindes?*
- *Gibt es etwas, was Sie besonders gut in Erinnerung haben (positive Momente / Situationen)? Gibt es etwas, was Sie eher schlecht in Erinnerung haben (negative Momente / Situationen)?*
- *Was würden Sie sich anders wünschen? Was könnte man verbessern? Welche Bedürfnisse haben Sie in diesem Bereich?*

**F: Lebenssituation der Familie**

**Welchen Effekt hat die Autismus-Spektrum Störung Ihres Kindes auf Ihre Familie und Sie Selbst?**

1. Situation Alltagsbewältigung
2. Eigene Erzählung

- *Wie bewältigen Sie Ihren Alltag? Haben Sie gewisse Bewältigungsstrategien entwickelt?*
- *Wie organisieren Sie sich als Familie? Wie ist die Aufgabenverteilung in Ihrer Familie? Wie ist der Zusammenhalt in Ihrer Familie? Haben Sie gewisse Routinen/Rituale?*
- *Haben Sie Ressourcen, die Sie unterstützen/entlasten? Familie, Freunde, Verwandte, Institutionelle Angebote*

1. Bewertung der Eltern

- *Wie zufrieden sind Sie im Allgemeinen mit Ihrer Situation?*
- *Was sind positive Momente für Sie als Familie? Was sind negative Momente für Sie als Familie?*
- *Was würden Sie sich anders wünschen? Was könnte man verbessern? Welche Bedürfnisse haben Sie in diesem Bereich?*
- *Wie zufrieden sind Sie im Allgemeinen mit der Situation zu Hause? Was ist belastend, was ist unterstützend? (auch ausserfamiliäre Ressourcen/Stressoren)*

**G Bezug zum Fragebogen**

Mögliche Folge-/Vertiefungsfragen:

- *Im Fragebogen, den wir Ihnen vor einigen Monaten geschickt hatten, haben Sie […] so beantwortet. Dazu würde mich noch interessieren, ob/wie […]*
- *Können Sie mir ein Beispiel nennen? In welchen Situationen war dies der Fall?*
- *Wie hat sich das auf Sie, Ihr Kind, Ihre Familie und Ihre Situation ausgewirkt?*
- *Möchten Sie zu einer Frage noch etwas anmerken/ ergänzen?*

| **Abschluss** |
| --- |

- *Wir sind am Ende des Interviews angelangt. Möchten Sie noch etwas hinzufügen, das für Sie wichtig ist/war und bis jetzt noch nicht angesprochen wurde?*
- *Ich möchte Ihnen gerne das Schlusswort überlassen: Was erscheint Ihnen am Wichtigsten, was möchten Sie gerne noch einmal wiederholen?*
- *Haben Sie noch Fragen oder Anmerkungen?*

__________________________________________________________________________________________________________________________________________________________________________________________________________________________________________________________________________________________________________________________________________________________________________________

Abschluss und Dank

*Dann bedanke ich mich ganz herzlich bei Ihnen für Ihre Offenheit und die Zeit, die Sie sich genommen haben!*

*Wenn Sie dies wünschen, erhalten Sie nach Abschluss der Arbeit gerne Einblick in die Ergebnisse* [E-Mail-Adresse aufnehmen!].
