# Supplementary material for: Autism spectrum: parents’ perspectives reflecting the different needs of different families
Source: BMC Pediatr. 2024 Jul 9;24:439. doi: 10.1186/s12887-024-04912-x (PMC11232266; doi:10.1186/s12887-024-04912-x)
Supplement: Supplementary file 2 — Supplementary Material 2 [file 12887_2024_4912_MOESM2_ESM.docx]

**Kategoriensystem Leitfadeninterview**

| **1.** | **Oberkategorie: Entwicklung des Kindes** | | | |
| --- | --- | --- | --- | --- |
|  | **Kategorie** | **Definition** | **Ankerbeispiel** | **Nr.** |
| 1.1 | Auffälligkeiten in der frühen kindlichen Entwicklung | Elterliche Einschätzung der Entwicklung ihres Kindes aufgrund ihrer Beobachtungen im Alltag (Frischknecht, Reimann und Grob, 2015). Die Manifestation sowie die Entwicklungsstörungen sind sehr unterschiedlich. Es gibt aber gewisse frühe Warnzeichen meist zwischen 12 und 24 Monaten (Zwaigenbaum, 2015). | «Genau betrachtet hat alles später angefangen, gekrabbelt ist er später. (-) Das mit dem Blickkontakt war eigentlich nicht so bei ihm aber vor allem die Spielentwicklung war im Vergleich zu Gleichaltrigen anders. Er hat anders gespielt. Zum Beispiel hat er immer wieder das Telefonkabel hin und her geschoben, solche Geschichten.» | B09, 20 |
| 1.2 | Elterliche Gedanken über Gründe der kindlichen Entwicklung | Gedanken zu Ursachen und Begründungen, welche sich die Eltern zur Entwicklung ihres Kindes machen (induktiv). | «Vom Umfeld sind wir (-), von der Familie her haben sie gefunden, dass wir sie falsch erziehen würde, das sei der Grund. Dann haben wir halt uns kritisch hinterfragt.» | B08, 10 |
| 1.3 | Verhaltensauffälligkeiten im Verlauf | Eltern berichten von unterschiedlichen Verhaltensauffälligkeiten ihrer Kinder im Verlauf (induktiv). | «Sie ist herumgesprungen, hat herumgeschrien bei jeder kleinsten Veränderung. Wie erwähnt hatte sie x Ausbrüche und sehr schwierig führbar.» | B08, 14 |
| 1.4 | Fortschritte | Eltern berichten von unterschiedlichen Fortschritten in der Entwicklung Ihres Kindes (induktiv). | «Zusammengefasst, wo er auf jeden Fall sich sehr, wo er grosse Schritte gemacht hat, war da in der Motorik. Er macht ja auch Ergotherapie, Psychomotorik. Natürlich er hat auch noch ein wenig Zerebralparese, er hat schon noch seinen kleinschrittigen Gang und ist schon eher der Grobmotoriker. Aber da hat er schon gut vorwärts gemacht auch so mit (-) schneiden und so. Ein wenig Feinmotorik, da hat er auf jeden Fall grosse Fortschritte gemacht.» | B09, 32 |

| **2.** | **Oberkategorie: Abklärungsprozess** | | | |
| --- | --- | --- | --- | --- |
|  | **Kategorie** | **Definition** | **Ankerbeispiel** | **Nr.** |
| 2.1 | Erleben des Abklärungsprozesses | Elterliches Erleben des Abklärungsprozesses (induktiv). | «Es war einfach eine sehr, sehr schwierige Phase. Es war eigentlich die schlimmste Phase, die wir hatten mit unseren Kindern.» | B03, 24 |
| 2.2 | Elterliche Reaktion auf Diagnose | Die Diagnose führt bei den Eltern zu unterschiedlichen Gefühlslagen wie Erleichterung, Trauer, Verlust, Schock und Überraschung sowie Selbstbeschuldigung (Hutton, 2005). | «Ich muss sagen, dass es dann für meinen Mann und mich, als er dann schliesslich doch diese ASS Diagnose erhalten hat, für uns war das eine totale Erleichterung. Ich hatte mich gedanklich aber auch schon lange damit arrangiert. Für uns war es eine Befreiung. Es gingen Türen auf, auch finanzieller Art.» | B10, 22 |
| 2.3 | Herausforderungen beim Abklärungsprozess | Eltern berichten von unterschiedlichen Belastungen während der Zeit des Abklärungsprozesses (induktiv). | «Ich kann mich nicht mehr so erinnern. Ich habe einfach die Erinnerung, dass ich da ziemlich alleine war irgendwie. Es war wirklich sehr anstrengend, irgendwie ging es dann weiter aber es wirklich sehr streng.» | B03, 28 |
| 2.4 | Ressourcen beim Abklärungsprozess | Eltern berichten von unterschiedlichen Ressourcen während der Zeit des Abklärungsprozess (induktiv). | «Das ist eigentlich das, diese guten, guten Tipps. Auch die Psychologin, die Logopädin, die unser Sohn am Anfang hatte, das waren für mich ganz wichtige Personen. Es war dann wie so eine Kette, die sich so aneinander reite.» | B10, 26 |
| 2.5 | Elterliche Beurteilung des Abklärungsprozesses | Elterliche Zufriedenheit mit dem Abklärungsprozess (Goin-Kochel, 2006; Chamak, 2011, Karst, 2012). | «Den Abklärungsprozess fand ich total gut. Also den ersten Test (-) fand ich schon sehr interessant. Ich habe immer Frau Dr. Schaefer angeschaut, die eine ganz warme Art hatte und wo unser Sohn sich sofort darauf eingelassen hat. Als wir dann da waren, da habe ich mich so aufgehoben gefühlt.» | B10, 22 |
| 2.6 | Elterliche Bedürfnisse Abklärung | Elterliche Bedürfnisse im Bereich der Abklärung (induktiv). | «Ich würde mir wünschen für andere Generationen die kommen, dass diese eine Begleitperson erhalten. Es ist vielleicht viel verlangt, aber ich denke diese benötigen, dass unbedingt, dass jemand ihnen beisteht, der alles weiss, alles drum herum, und diese Familien dann auch begleitet.» | B01, 38 |

| **3.** | **Oberkategorie: Therapeutische Massnahmen** | | | |
| --- | --- | --- | --- | --- |
|  | **Kategorie** |  | **Ankerbeispiel** | **Nr.** |
| 3.1 | Erleben der Therapien | Elterliches Erleben der verschiedenen Therapien (induktiv). | «Nein, also wirklich alles top Profis. Nicht nur fachlich, sondern auch auf der persönlichen Ebene und das schätzten wir sehr. Sie haben uns wirklich geholfen und unterstützt. Sie haben sich um uns als Personen und um uns als Familie gekümmert. Das war wirklich sehr positiv. Das war wirklich bestens.» | B06, 28 |
| 3.2 | Elterlicher Aussagen bezüglich autismusspezifischer Therapien | Eltern haben unterschiedliche Standpunkte bezüglich autismusspezifischer Therapien (induktiv). | «Ich habe dann im Nachhinein gemerkt, dass es so Intensivtherapien gäbe, so x Stunden pro Wochen in Zürich und so. Da wurden wir nicht darüber informiert aber das ist mir auch ganz recht. Weil das hätten wir sowieso nicht gewollt.» | B08, 40 |
| 3.3 | Elterliche Beurteilung Therapien inkl. Intensität und Distanz | Die Beurteilung der Zufriedenheit der Eltern mit den genutzten pädagogischen und therapeutischen Massnahmen im Vorschulalter (Eckert, 2015; Bitterman, 2008). | «Also mit den Therapien vor dem Kindergarten war ich voll und ganz zufrieden. Wie gesagt, nicht nur fachlich, sondern auch auf der persönlichen Ebene.» | B06, 36 |
| 3.4 | Bedürfnisse Therapien | Angaben von zusätzlich erwünschten Therapieangeboten (induktiv). | «Wenn es unendliche Ressourcen aller Art geben würde, auch finanziell. Dann hätte ich mir gewünscht, dass unsere Tochter mehr Therapiestunden gehabt hätte. Dass aber dann mit einer Begleitperson, die sie in die Therapien und auch wieder zurückbegleitet, sei es zum Beispiel mit dem Taxi.» | B06, 42 |

| **4.** | **Oberkategorie: Bildungsweg** | | | |
| --- | --- | --- | --- | --- |
|  | **Kategorie** | **Definition** | **Ankerbeispiel** | **Nr.** |
| 4.1 | Erleben der Schulbildung | Elterliches Erleben der Schulbildung (induktiv). | «Es funktioniert gut. Das einzige, was jetzt (-) natürlich nicht so ideal ist, ist die soziale Interaktion mit den anderen Kindern. Also er kommt jetzt nicht so gross ins Spiel mit ihnen, aber er wird akzeptiert. Er ist Bestandteil der Gruppe, aber es ist jetzt natürlich nicht so, dass er da jetzt ständig mit ihnen im Spiel ist.» | B09, 90 |
| 4.2 | Beratung bezüglich geeigneter Schulform | Elterliche Beurteilung der Beratung bezüglich geeigneter Schulform (induktiv). | «Eben die Heilpädagogin, die wir hatten. Sie hat mich sehr gut beraten. Bei ihr habe ich gewusst, dass sie mir nicht irgendetwas angibt, was der Schule guttut, so blöd gesagt, sondern etwas, was einfach unserem Sohn hilf und für ihn gut ist.» | B13, 60 |
| 4.3 | Kommunikation mit den Lehrpersonen | Elterliche Einschätzung der Kommunikation sowie des Informationsaustausches mit den Lehrpersonen in der Schule (Renty, 2006; Rattaz, 2014). | «Ich habe einen engen Kontakt mit den Lehrpersonen. Mit der Lehrerin spreche ich jeden Freitagnachmittag nach der Schule (-) und sie gibt mir ein Feedback, wie die Woche für unseren Sohn war, was positiv war oder was nicht so gut war.» | B07, 54 |
| 4.4 | Elterliche Aussagen bezüglich Schulform (integrativ vs. separativ) | Es sind sowohl Befürworter des integrativen Schulsettings sowie auch Verfechter einer schulischen Förderung in Sonderschulen anzutreffen (Eckert, 2012) | «Nachher in die erste Klasse, da hatte ich schon grosser Respekt. Alle die ich kenne, haben ihre Kinder in der HPS, ich bin glaube ich bin die einzige, die ich kenne, deren Sohn in die Schule geht. Von den Erzählungen von den anderen, war ich immer etwas skeptisch.» | B13, 56 |
| 4.5 | Elterliche Beurteilung der Schulform | Elterliche Beurteilung bezüglich der Zufriedenheit der Schulbildung ihres Kindes (Spann, 2003). | «Sehr, sehr zufrieden. Wenn ich eine Zahl sagen müsste und das höchste wäre eine 10, dann wäre es eine 10. Mein Wunsch war, dass er sein "Plätzli" findet und er hat wirklich seinen Platz gefunden.» | B04, 62 |
| 4.6 | Bedürfnisse Schulbildung | Elterliche Bedürfnisse im Bereich der Schulbildung (induktiv). | «Was ich schon noch cool fände in der Regelschule, wäre wenn man ein bisschen mehr Fachkompetenzen hätte.» | B12, 100 |
| 4.7 | Auswirkungen Corona auf Schule | Elterliches Erleben der Schule unter Corona-Bedingungen (induktiv) | «Ich habe das schon gemerkt in dieser Zeit im Lockdown, wo ich mit Homeschooling gemacht habe und er da seine Leistung gezeigt hat, da war er um einiges besser, würde ich sagen. Schon wahrscheinlich in einigen Bereichen sogar überdurchschnittlich. Aber in der Schule ist er glaube ich zu abgelenkt.» | B09, 32 |

| **5.** | **Oberkategorie: Freizeitgestaltung des Kindes** | | | |
| --- | --- | --- | --- | --- |
|  | **Kategorie** | **Definition** | **Ankerbeispiel** | **Nr.** |
| 5.1 | Erleben der Freizeitgestaltung | Elterliches Erleben der Freizeitgestaltung (induktiv). | «Dort sehen wir uns wirklich beschränkt. (-) Hier ist nochmals ein wenig das Gleiche. Für viele Aktivitäten muss man die Kinder begleiten, 1:1. Wie macht das meine Frau, wenn sie gleichzeitig noch ein anderes Kind betreuen muss? Wie macht man das?» | B06, 58 |
| 5.2 | Ressourcen Freizeit | Erwähnte Ressourcen im Bereich der Freizeit (induktiv). | «Meine Mutter ist mir hier eine grosse Hilfe. Also es ist schon so, dass meine Mutter auch am Wochenende ihn nimmt, in die Berge geht, weil wir das einfach nicht können.» | B11, 65 |
| 5.3 | Elterliche Beurteilung Freizeit | Elterliche Beurteilung der Freizeitgestaltung ihres autistischen Kindes, wobei diese eher als negativ beurteilt wird (Walton, 2019). | «Dort bin ich wenig zufrieden ehrlich gesagt. Ich weiss nicht, eine 4 oder 5 von 10. Hier gibt es Verbesserungspotential.» | B06, 62 |
| 5.4 | Elterliche Bedürfnisse Freizeit | Elterliche Bedürfnisse im Bereich der Freizeitgestaltung (induktiv). | «Unser Sohn würde gerne irgendwo in eine Sportgruppe gehen. Es muss ja im Prinzip ein Trainer sein, der sich mit uns auskennt und auf ihn eingehen kann. Es wäre einfach schön, wenn es so ein Angebot gäbe.» | B02, 88 |
| 5.5 | Auswirkung Corona auf Freizeitgestaltung | Elterliches Erleben der Freizeit unter Corona-Bedingungen (induktiv). | «Eine andere Mutter sagte einmal zu mir, ob sie meinen Sohn nicht einmal zum Schachkurs mitnehmen soll, sie könnte sich vorstellen, dass das total etwas für ihn wäre. Da hält mich im Moment Corona sehr zurück. Da bin ich sehr vorsichtig.» | B10, 52 |

| **6.** | **Oberkategorie: Lebenssituation der Familie** | | | |
| --- | --- | --- | --- | --- |
|  | **Kategorie** | **Definition** | **Ankerbeispiel** | **Nr.** |
| 6.1 | Auswirkungen auf die Familie | Auswirkungen der ASS auf die Familie (Eltern, Geschwister) inkl. erweiterter Familie (Myers, 2009). | «Sonst auf uns als Familie, hat es schon einen riesen Einfluss. Wenn ich jetzt mit der Zeit vorher vergleiche, die drei Jahre, als wir nur den älteren Sohn hatten (-), wow, was könnten wir für Reisen machen, wie viel Zeit hätten wir als Eltern für uns.» | B10, 68 |
| 6.2 | Externe Betreuung des Kindes | Professioneller Dienst, durch welchen das Kind regelmässig betreut wird, wie beispielsweise eine Tagesmutter oder eine Kindertagesstätte, welche ohne Wertung genannt werden (induktiv). | «Sonst haben wir noch jemanden vom Entlastungsdienst für unseren Sohn, sie kommt jetzt seit fast 3 Jahren 2 Stunden pro Woche. Dann haben wir noch die Kinderspitex seit Dezember.» | B05, 6 |
| 6.3 | Ressourcen | Verfügbare Ressourcen auf familiärer Ebenen (Petermann und Schmidt, 2006) sowie aus der sozioökonomischen Umgebung wie Verwandte, Freunde, andere betroffene Familien und Fachpersonen welche von den Eltern als positiv gewertet werden (Gray, 2006; Twoy, 2007) | «Meine Schwester hauptsächlich. (-) Meine Schwester. Sie ist (-) ganz anders als, älter als ich, also nur 3 Jahre älter, ist auch in Deutschland. Aber wenn wirklich alle Stricke reissen, dann spreche ich eigentlich immer mit meiner Schwester, die hat eine ganz pragmatische Herangehensweise (lacht).» | B10, 72 |
| 6.4 | Bewältigungsstrategien | Eltern von Kindern mit einer ASS nutzen unterschiedliche Bewältigungsstrategien (Hastings, 2005; Gray, 2006; Twoy, 2007). | «Ich kann mich glaube ich auch sehr (-) daran freuen, ich kann mich sehr daran freuen, auch an diesen positiven Sachen, die ich sonst mit einem anderen Kind gar nicht erleben würde.» | B10, 70 |
| 6.5 | Routinen/Rituale | Familien mit einem Kind mit einer ASS haben unterschiedliche Routinen/Rituale (induktiv). | «Ich habe schon ganz früh immer einen Wochenplan erstellt mit Magneten. Das man so gewisse Dinge, wie er geht zur Tagesmutter, drauf machte oder Therapie oder später dann die Schule. Ich habe auch sehr früh begonnen mit Piktogrammen zu arbeiten, mit Plänen.» | B02, 100 |
| 6.6 | Isolation | Soziale Isolation der Eltern aufgrund des erhöhtem Organisationsbedarf sowie befürchteter Reaktionen des Umfeldes (Woodgate, 2008; Ludlow, 2012). | «Ich fühle mich so isoliert. Ich habe wie gesagt ein paar Freundinnen verloren auf diesem Weg.» | B07, 58 |
| 6.7 | Reaktion Umfeld | Eltern von Kindern mit ASS erfahren häufig wenig Verständnis der Umgebung, was sich durch Vermeidungsverhalten, feindlichem Anstarren sowie unhöflichen Kommentaren äussert (Gray, 2002). | «Vom Freundeskreis her kann ich jetzt nicht so sagen, da habe ich doch das Gefühl, dass manche Freundschaften sich sehr entfernt haben, weil Treffen mit unserem Sohn nun einmal ein bisschen anders wären und es können sich nicht alle darauf einlassen.» | B10, 26 |
| 6.8 | Finanzielle Situation | Eltern von Kindern mit einer ASS erleben eine finanzielle Belastung, welche durch erhöhte Kosten für Therapien oder Institutionen und verminderten Einnahme entsteht (Myers, 2009). | «Am Ende hat man auch finanziell eine dermassen grosse Belastung, weil du eigentlich nur noch am Zahlen bist.» | B11, 17 |
| 6.9 | Positive Momente als Familie | Momente, welche von den Eltern als positiv bewertet werden (induktiv). | «Wir haben schon sehr viele schöne Erlebnisse zusammen in der Natur, beim Velofahren, im Wald, beim Wandern in der Badi, in den Ferien. Beim Laufen ist es dann oft auch so, dass wir sie aufteilen können. Es kommt auch so eine andere Stimmung rein, wenn man so am Laufen ist, dann ist man so im Fluss.» | B03, 104 |
| 6.10 | Schwierige Momente als Familie | Momente, welche von den Eltern als negativ bewertet werden (induktiv). | «Ja eben, wenn er manchmal so ausrastet. (-) Wenn es wie eine Reizüberflutung ist, dann kann er mittlerweile schon sehr Gas geben. Also ersten hat er ziemlich Kraft, also nicht nur ziemlich, er hat viel Kraft.» | B13, 92 |
| 6.11 | Elterliche Beurteilung Ihrer Lebenssituation | Elterliche Beurteilung bezüglich der Zufriedenheit Ihrer Lebenssituation (induktiv). | «Aber ansonsten würde ich wirklich fast sagen, bin ich eigentlich wunschlos glücklich. (--) Ich glaube, wir haben sehr viel Glück. Wir können uns nicht beschweren über unser Leben.» | B10, 82 |
| 6.12 | Elterliche Bedürfnisse Lebenssituation | Elterliche Bedürfnisse im Bereich der Lebenssituation (induktiv). | «Für mich ist das Wichtigste der Unterstützungsfaktor (-) zu Hause, in privater Natur und auch in der Schule. Ich finde, da gibt es noch ein sehr grosses Potential.» | B11, 101 |
| 6.13 | Auswirkung Corona auf Lebenssituation | Elterliches Erleben der Lebenssituation unter Corona-Bedingungen (induktiv). | «Ja also eben, am Wochenende war die Ressource, dass meine Eltern auf ihn aufgepasst haben, wenn mein Mann und ich einmal was Schönes unternommen haben. Das ist jetzt seit einem Jahr halt nicht, dass merkt man schon. Das merke ich schon langsam. Ich habe jetzt weniger Zeit mit meinem Mann zu zweit, weil er natürlich auch immer am Wochenende mit uns zusammen ist.» | B09, 118 |
| 6.14 | Feedback Interview | Elterliches Feedback der Eltern bezüglich des Interviews (induktiv) | «Ich finde es toll, dass sie sich interessieren und auch diese Interviews machen dürfen. Ich finde es ganz "lässig", das sich andere Leute interessieren und mehr erfahren möchten. Nur dank so Menschen wie sie, die das Ganze auch etwas nach aussen tragen können. Sie sammeln wie so die Stimmen von uns (lacht). Ich finde das so etwas Schönes und Tolles.» | B04, 96 |
